# Regressed three-dimensional capillary network and inhibited angiogenic factors in the soleus muscle of non-obese rats with type 2 diabetes

**DOI:** 10.1186/1743-7075-8-77

**Published:** 2011-11-03

**Authors:** Hiroyo Kondo, Hidemi Fujino, Shinichiro Murakami, Fumiko Nagatomo, Roland R Roy, Akihiko Ishihara

**Affiliations:** 1 Department of Food Science and Nutrition, Nagoya Women's University, Nagoya, Japan; 2 Department of Rehabilitation Science, Kobe University Graduate School of Health Sciences, Kobe, Japan; 3 Laboratory of Cell Biology and Life Science, Graduate School of Human and Environmental Studies, Kyoto University, Kyoto, Japan; 4 Brain Research Institute, University of California, Los Angeles, California, the USA; 5 Department of Integrative Biology and Physiology, University of California, Los Angeles, California, the USA

**Keywords:** angiogenic factors, capillary network, skeletal muscle, three-dimensional imaging, type 2 diabetes

## Abstract

Based on findings obtained using two-dimensional capillary analyses on tissue cross-sections, diabetes has been shown to be associated with a high risk for microangiopathy and capillary regression in skeletal muscles. We visualized the three-dimensional architecture of the capillary networks in the soleus muscle of non-obese Goto-Kakizaki (GK) rats with type 2 diabetes and compared them with those of control Wistar rats to provide novel information, e.g., capillary volume, on the capillary networks. In addition, we examined pro- and anti-angiogenic gene expression levels in the soleus muscle of GK rats using TaqMan probe-based real-time PCR. As expected, plasma glucose levels were higher and insulin levels lower in GK than control rats. The three-dimensional architecture of the capillary networks was regressed and capillary volume was smaller in the soleus muscle of GK compared to control rats. The mRNA expression levels of the pro-angiogenic factors HIF-1α, KDR, Flt-1, ANG-1, and Tie-2 were lower, whereas the level of the anti-angiogenic factor TSP-1 was higher in GK than control rats. These data suggest that a decrease in pro-angiogenic and increase in anti-angiogenic factors may play an important role in type 2 diabetes-induced muscle circulatory complications.

## Background

Skeletal muscle homeostasis and function are dependent on adequate blood flow, oxygen delivery, and substrate exchange at the capillary level. Microvascular complications are major risk factors associated with diabetes [1]. The degenerative alterations in the capillary networks observed in muscles of diabetic subjects impair blood flow, oxygen delivery, and substrate exchange and can lead to serious conditions, such as diabetic foot ulcers, gangrene, and amputation [2]. Although it is known that skeletal muscles in animal and human subjects with type 1 or 2 diabetes have impaired angiogenesis and a reduced capillary network [3-5]based on two-dimensional analyses on tissue cross sections. No data are available from three-dimensional capillary analyses that would provide novel information, such as changes in capillary volume. Furthermore, the possibility that changes in the levels of angiogenic factors may be associated with diabetes-related microangiopathy has not been thoroughly examined. This possibility could involve multifactorial processes, i.e., changes in the levels of pro-angiogenic as well as anti-angiogenic factors in the skeletal muscles of diabetic subjects. Therefore, the purpose of the present study was to investigate the changes in three-dimensional capillary network and multifactorial angiogenic gene expression levels of the soleus muscle in non-obese Goto-Kakizaki (GK) rats with type 2 diabetes.

## Methods

The present experiments were conducted in accordance with the National Institutes of Health (NIH) Guide for the Care and Use of Laboratory Animals (National Research Council, 1996) and approved by the Institutional Animal Care and Use Committee of Kobe University, Japan.

**Figure 1:**
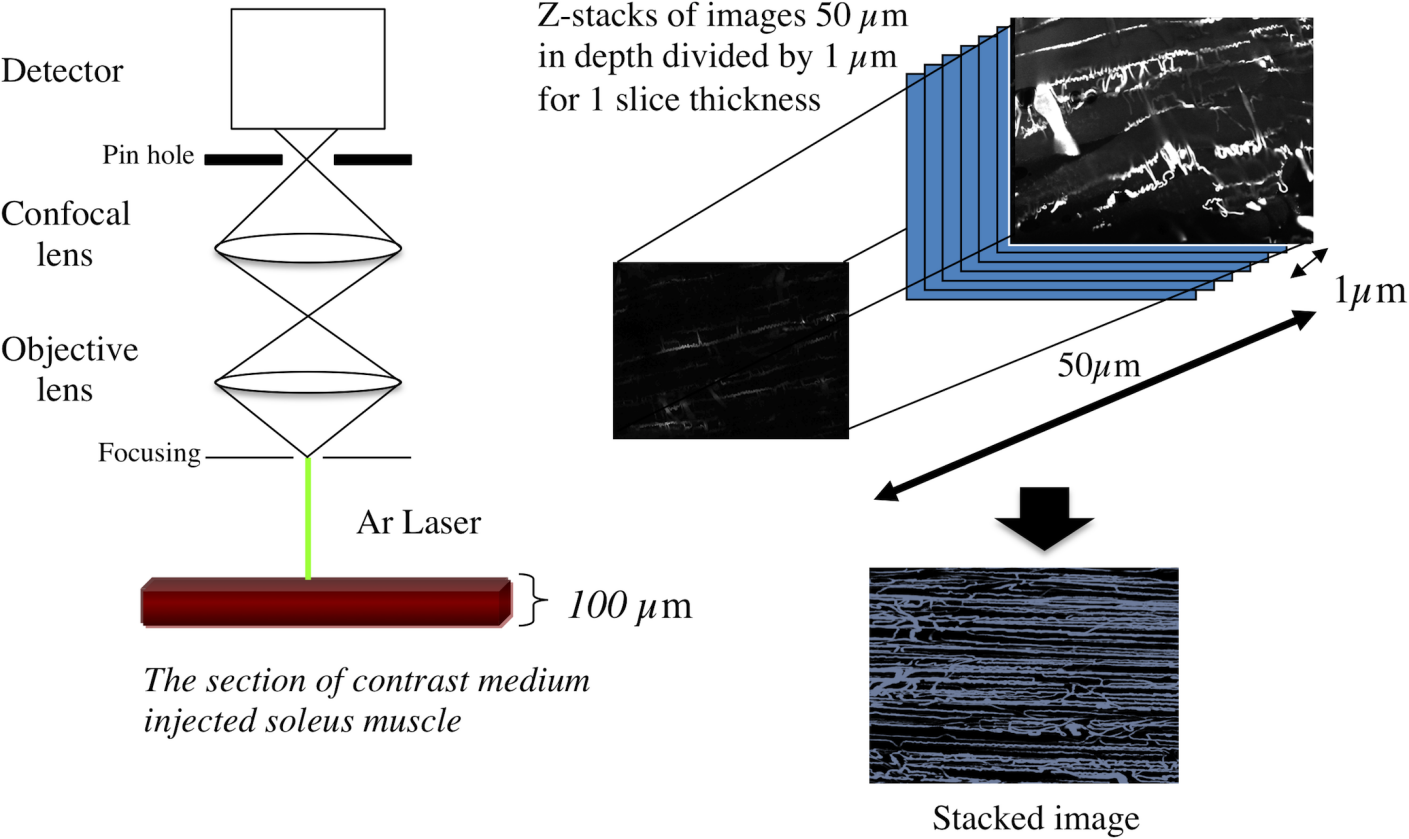
**A schematic of the three-dimensional visualization and capillary structural analyses is shown**. Confocal laser scanning microscopy was used to visualize the three-dimensional capillary architecture. Longitudinal microscopic images from the mid-belly and middle portion of the soleus muscle were obtained at a magnification of x20 and scanned for a depth of 50 μm at a 1-mm slice thickness.

Nine-week-old male non-obese diabetic GK and non-diabetic control Wistar rats (*n *= 6 in each group) were used in the present study. After blood sampling from the heart under anaesthesia (pentobarbital, 5 mg/100 g, *i.p.*), the left soleus muscle was removed, weighed, and stored in RNA stabilization solution (~10 mg) or in the freezer at -80°C (remainder of the muscle). The right soleus muscle was perfused with a physiological sodium chloride solution followed by the infusion of contrast medium including 8% gelatin and 1% fluorescent material (PUSR80, Mitsubishi Pencil, Tokyo, Japan). Thereafter, the muscle was removed, quick-frozen in isopentane pre-cooled in liquid nitrogen and stored at -80°C until further analyses. Plasma glucose, and insulin were measured. The capillaries in the soleus muscle were traced using a confocal laser microscope (TCS-SP5, Leica, Germany) using procedures described in detail previously [6]. Briefly, longitudinal microscopic images from the mid-belly of the soleus muscle were obtained at a magnification of x20 and scanned for a depth of 50 μm at a 1-mm slice thickness. The images were rendered automatically and displayed as three-dimensional images (Figure 1). The number of capillaries was counted and the capillary volume was calculated by summing the capillary areas on corresponding planes.

Total RNA was extracted from approximately 10 mg of each muscle by an extraction kit (QuickGene RNA tissue kit SII, Fujifilm, Japan). The expression levels of the pro-angiogenic factors VEGF (Rn00582935_m1), KDR (Rn00564986_m1), Flt-1 (Rn00570815_m1), angiopoietin-1 (ANG-1, Rn00585552_m1), angiopoietin-2 (ANG-2, Rn01756774_m1), Tie-2 (Rn01433337_m1), HIF-1α (Rn00577560_m1) and its anti-angiogenic factor, and thrombospondin-1 (TSP-1, Rn01513693_m1) then were quantified by TaqMan gene expression assays using real-time PCR (7500 Fast, Applied Biosystems). The housekeeping gene 18S was used as an internal standard.

Homogenized muscle protein samples were separated on SDS-PAGE and transferred to PVDF membrane. Western blots were visualized with enhanced chemiluminescence (Amersham Biosciences, Piscataway, NJ). The primary antibody used in this study was an anti-VEGF at a dilution of 1:200 (sc-7269; Santa Cruz Biotechnologies, Santa Cruz, CA). The density of each band for both groups was divided by the average density of the control group and compared.

All data are presented as mean ± SEM. Group differences in glucose levels, insulin levels, capillary volume, or expression of angiogenic factors between non-diabetic and diabetic rats were assessed by Student *t*-tests. For all analyses *P *< 0.05 was considered as significant.

## Results

**Figure 2:**
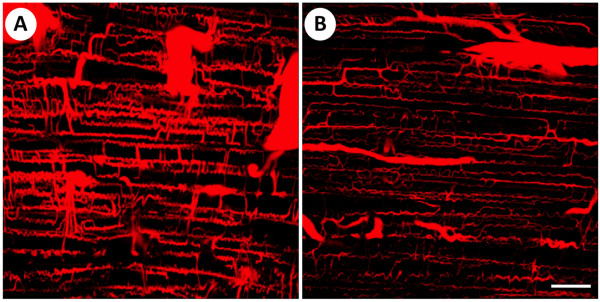
**Images of the capillary network of the soleus muscle in control (A) and GK (B) rats obtained by confocal laser scanning microscopy**. Image depth (z-axis) is 50 μm. Note the lower amount of staining in the GK vs. Control group, reflecting a lower capillary volume in the GK group. Scale bar in (B) = 100 μm and is the same for both panels.

**Figure 3:**
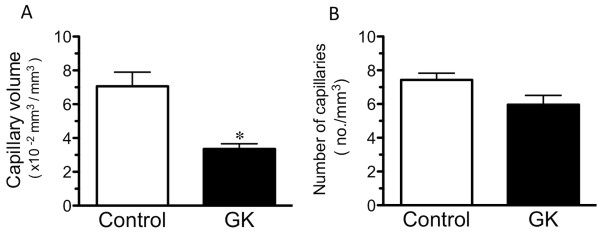
**The mean (*n *= 6 rats/group) capillary volumes (A) and the number of capillaries (B) in the soleus muscle of the Control and GK groups determined using confocal laser microscopy**. The mean capillary volume was smaller in GK than Control group, whereas the mean number of capillaries was not different for the two groups. *, significantly different from the Control group at *p *< 0.05.

**Figure 4:**
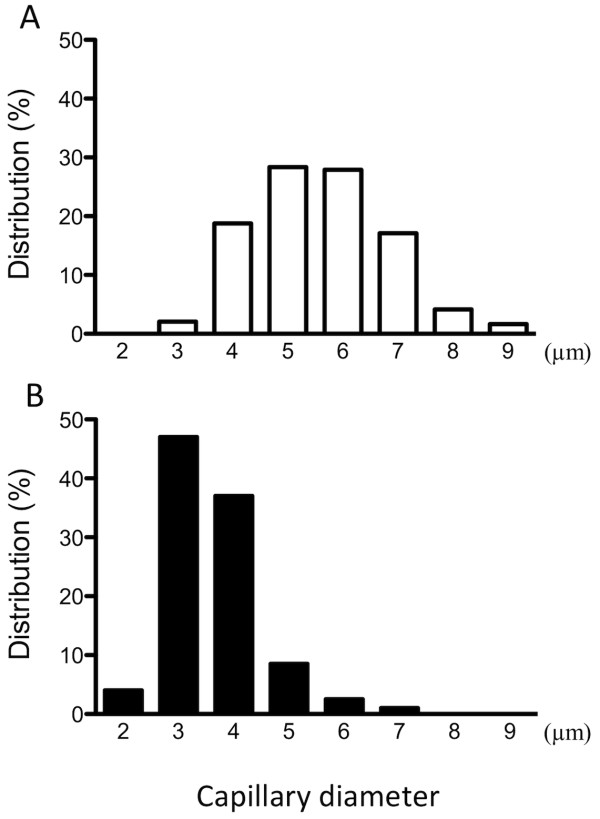
**Frequency distributions of the luminal diameters of the capillaries in the soleus muscle of the Control (A, open bars) and GK (B, closed bars) groups are shown**. Note that the histogram for the GK group is shifted to the left compared to the Control group, indicating a larger number of small capillaries in the GK than Control group.

Plasma glucose levels were higher and plasma insulin levels lower in GK than control rats (Table 1). Representative confocal three-dimensional images of the capillary network in the soleus muscle of control and GK rats are shown in Figure 2. Visually, the capillaries had a smaller diameter in GK than control rats. This observation is consistent with the mean muscle capillary volume being 47% lower in GK than control rats (Figure 3A). The number of capillaries in the soleus muscle was not different between the control and GK rats (Figure 3B). The mean luminal diameter of the capillaries, however, was larger in the control (5.1 ± 0.3 μm) than GK (3.1 ± 0.2 μm) group, as reflected in the shift to the left (smaller sizes) in the luminal diameters of the GK group (Figure 4).

**Table 1:** Plasma glucose and insulin levels of control and Goto-Kakizaki (GK) rats

	Control	GK
Glucose (mg/dL)	114 ± 7	270 ± 12*
Insulin (ng/mL)	2.8 ± 1.6	1.1 ± 0.2*

Values are means ± SEM, *n *= 6 rats/group. *, significantly different from control at *p *< 0.05.

**Figure 5:**
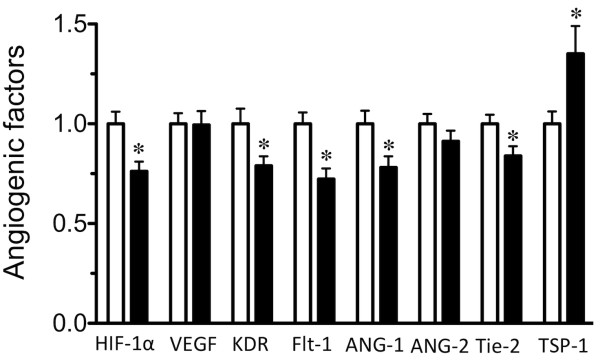
**The mean (SEM, *n *= 6 rats/group) mRNA expression levels for the angiogenic factors in the Control (open bars) and GK (filled bars) groups**. The values for the GK group are expressed as a fold-change (AU) from the values for the Control group that are set to 1. *, significantly different from Control group at *p *< 0.05.

**Figure 6:**
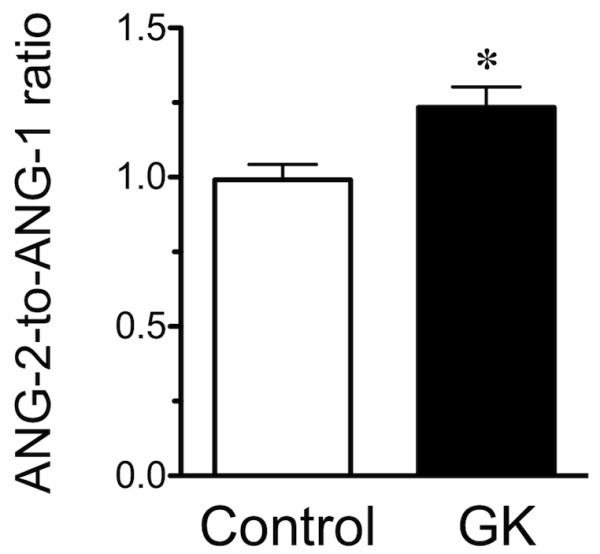
**The mean ANG-2-to-ANG-1 ratio in the Control and GK groups is shown**. *, significantly different from Control group at *p *< 0.05.

**Figure 7:**
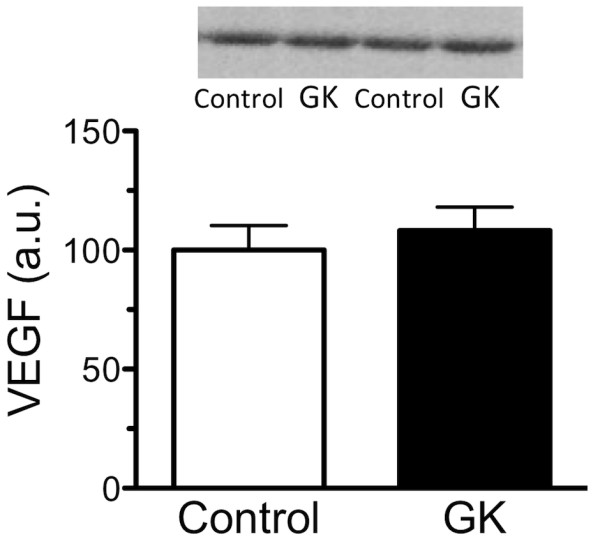
**The mean expression level of VEGF protein in the Control and GK groups is shown**. VEGF protein was not different between the two groups.

The mRNA expression levels of the pro-angiogenic factors HIF-1α, KDR, Flt-1, ANG-1, and Tie-2 were lower in the soleus muscle of GK compared to control rats, whereas the levels of VEGF and ANG-2 were unaffected (Figure 5). As a result, the ANG-2-to-ANG-1 ratio was higher in GK than control rats (Figure 6). In contrast, the mRNA expression level of the TSP-1, an anti-angiogenic factor, was higher in GK than control rats (Figure 5). Furthermore, VEGF protein was not different between the control and GK rats (Figure 7).

## Discussion

Using three-dimensional analyses visualized by confocal laser microscopy, we found a regression of the capillary networks in the soleus muscle of GK compared to control rats. Furthermore we provide novel information on a decreased capillary volume, lower levels of pro-angiogenic factors, and a higher level of an anti-angiogenic factor in the soleus muscle of GK compared to control rats. Combined, these results suggest that angiogenic factors may play a role in the observed impairment in the capillary networks in skeletal muscles in subjects with type 2 diabetes.

Based on two-dimensional capillary staining on muscle cross sections [3-5], diabetes-induced remodeling of the capillary networks in skeletal muscles results in a low capillary-to-fiber ratio and thus a longer capillary diffusion distance for metabolic exchange. Similarly, a low functional capillary density has been observed in skeletal muscles of Zucker diabetic fatty rats using *in vivo *microscopy [7]. Using three-dimensional analyses visualized by confocal laser microscopy, we now report to our knowledge for the first time a lower capillary volume in diabetic compared to control rats.

A balance between pro-angiogenic and anti-angiogenic factors modulates angiogenesis. Kivela et al. [4] reported that many of the genes known to increase angiogenesis, i.e., VEGF and their receptors, were lower in subjects with type 1 diabetes than in control. In addition, the levels of TSP-1 and Retinoblastoma-like 2, i.e., factors known to inhibit angiogenesis in skeletal muscles, were higher in type 1 diabetic than control mice. In the present study, the levels of all pro-angiogenic factors studied, except VEGF and ANG-2, were lower in the soleus of GK compared to control rats (Figure 3). Furthermore, the ANG-2-to-ANG-1 ratio was higher in GK than control rats. The ANG-2-to-ANG-1 ratio is thought to determine whether the net effect of these angiopoietins is to stabilize or destabilize the vasculature, i.e., an elevated ANG-2-to-ANG-1 ratio induces destabilization of the vasculature resulting in capillary regression [8]. Finally, the expression level of TSP-1, an inhibitor of angiogenesis [9], was higher in GK than control rats. Thus all of these differences between the diabetic and control rats are consistent with the prominence of microangiopathy in the skeletal muscles of diabetic rats. In conclusion, the data indicate that a decrease in the levels of pro-angiogenic factors and an increase in anti-angiogenic factors may play an important role in the capillary regression associated with type 2 diabetes.

## List of Abbreviations

Flt-1: fms-like tyrosine kinase 1 or vascular endothelial growth factor receptor-1; GK: Goto-Kakizaki; HIF-1α: hypoxia-inducible factor 1alpha; KDR: kinase insert domain receptor or vascular endothelial growth factor receptor-2; PCR: polymerase chain reaction; Tie-2: tyrosine kinase with immunoglobulin-like and EGF-like domains or receptor tyrosine kinase; VEGF: vascular endothelial growth factor.

## Competing interests

HK, HF, SM, FN, RRR and AI have no relevant conflict of interest to disclose.

## Authors' contributions

All authors read and approved the final manuscript. HK, HF, and AI conceived the study and obtained funding. HK, HF, SM and NF coordinated the data collection. HK conducted the data analysis, and HK, HF, RRR and AI interpreted the results and wrote the manuscript.
